# A Ruptured Extra-dural Spinal Arterio-venous Malformation Presenting as Horner's Syndrome: The First Case Report

**DOI:** 10.7759/cureus.1645

**Published:** 2017-09-03

**Authors:** Sunil Munakomi

**Affiliations:** 1 Neurosurgery, Nobel Teaching Hospital, Nepal

**Keywords:** horner's, hematoma, spinal, arterio-venous malformation

## Abstract

We report the first case of a young patient who presented with the features of Horner's syndrome following a spinal extradural hematoma resulting from a ruptured arterio-venous malformation (AVM). Since there were features of early compressive myelopathy as well, urgent magnetic resonance imaging (MRI) scan of the spine revealed features suggestive of an extradural hematoma in the cervico-thoracic junction. The patient underwent an emergent laminectomy with an evacuation of the hematoma. The histological features were consistent with that of an AVM.

## Introduction

The initial presentation of a ruptured spinal extra-dural arterio-venous malformation (AVM) as Horner’s syndrome in a young male has not been reported in the literature to date. Extradural spinal hematoma accounts for only 5% of all reported spinal AVM cases at the initial presentation [1].

Here, we report the case of an extra-dural spinal hematoma resulting from a ruptured AVM in a young male patient. He presented with the features of Horner’s syndrome along with the presence of sensory dysesthesia along the C7 and T1 dermatomal distribution on his left arm. He also had findings suggestive of an early compressive myelopathy. He underwent emergent laminectomy with an evacuation of the hematoma following which he had a good neurological recovery. The histopathological report confirmed the diagnosis of an AVM.

## Case presentation

A 16-year-old male from Terathum, Nepal presented to our out-patient clinic with a history of a sudden onset of drooping of his left eye lid followed by an abnormal sensation along the medial aspect of his left arm and forearm that developed over the previous three days. He had mild pain in the nape of his neck while he was removing mud with a shovel preceding these symptoms. He did not report any loss of consciousness, weakness of his arms or legs, or any bladder or bowel incontinence. He had no other significant past medical or surgical illnesses. There was no history suggestive of any bleeding disorders and the blood investigation did not reveal coagulopathy.

**Figure 1:**
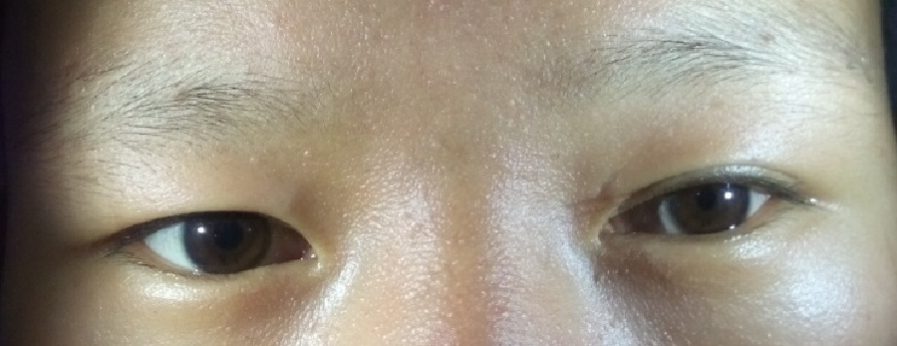
Left-sided ptosis and miosis

On neurological examination, the patient was conscious and oriented to time, place, and person. There was a mild drooping of his left eyelid with the presence of miosis on his left pupil (Figure 1).

**Figure 2:**
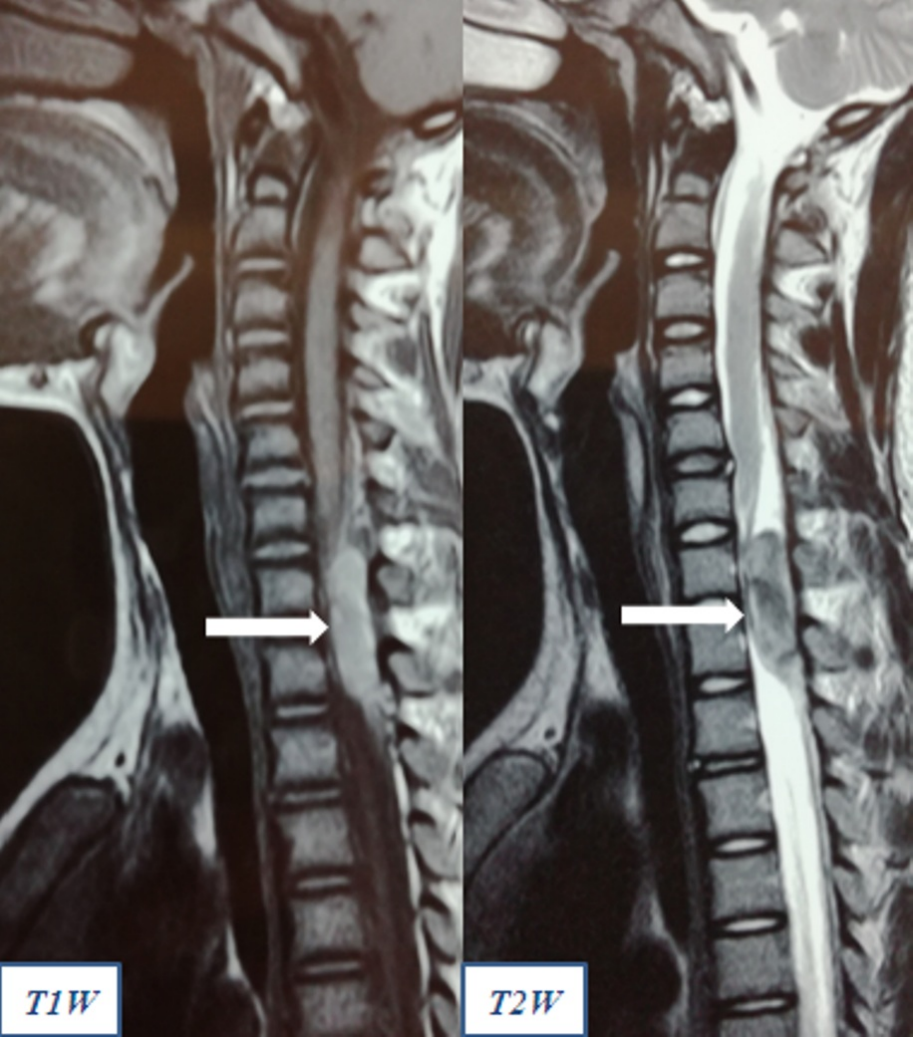
Magnetic resonance images suggestive of an extra-dural hematoma in the cervico-thoracic junction

There was sensory dysesthesia along the C7, C8, and T1 dermatomal distribution on his left arm. The motor power in his upper and lower limbs was normal. There was however, evidence of early myelopathy with regards to the presence of hypertonia in the muscles and increased deep tendon reflexes. The clinical localization and diagnosis was probably due to an acute onset of extra-dural compressive pathology in the C7 –T1 region. Immediate magnetic resonance imaging (MRI) scan of the spine revealed findings suggestive of an acute extra-dural hematoma in the C7-T1 region with lesion being hyper-intense in the T1 and hypo-intense in the T2 weighted images (Figure 2 ).

The patient was immediately taken up for a laminectomy and evacuation of the hematoma. Intra-operatively, there was the presence of multiple tangles of small vessels incorporating within the hematoma and arising from the nerve roots at the dural sleeves.

**Figure 3:**
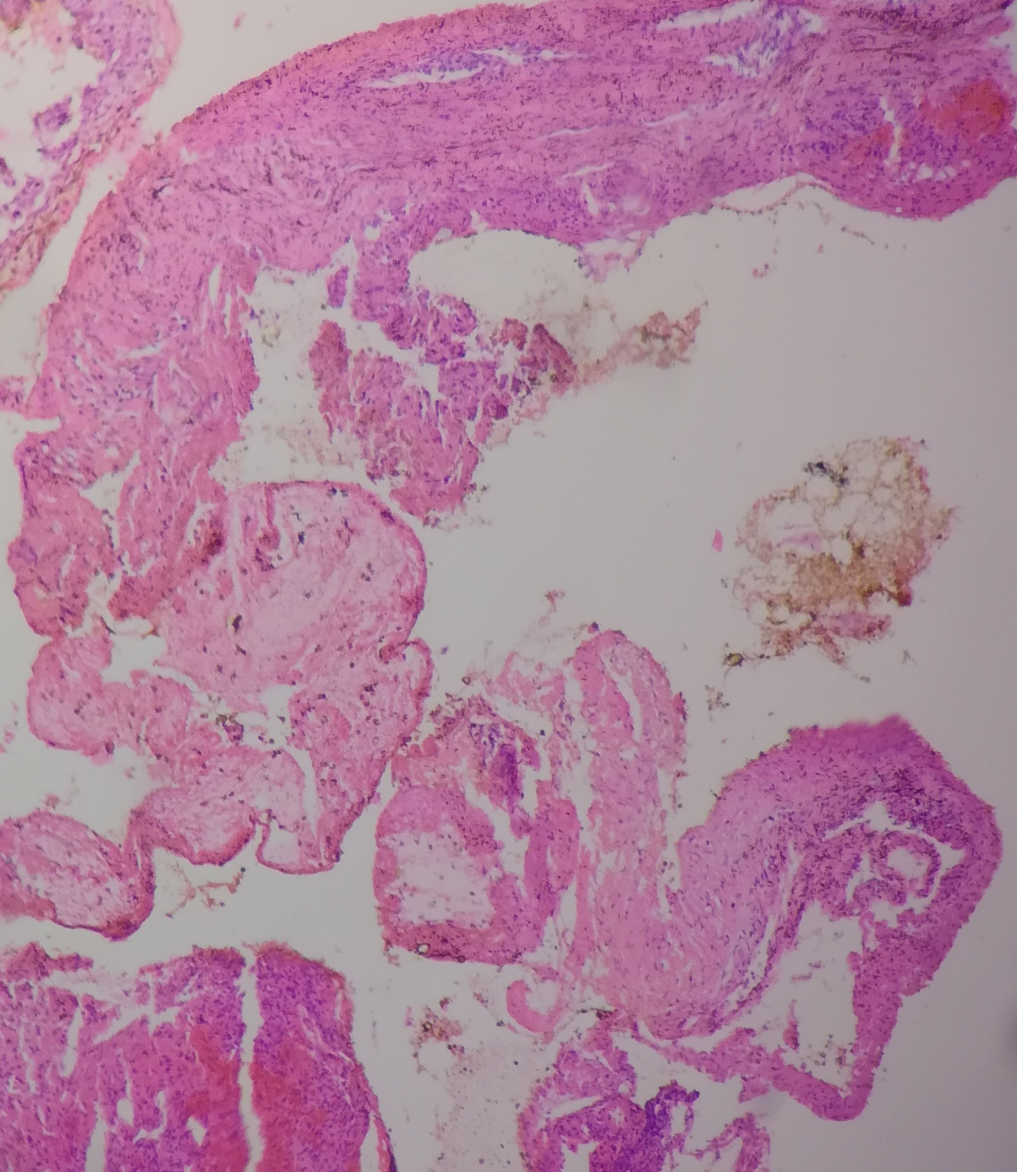
Histology findings suggestive of an arterio-venous malformation

Post-operatively, the patient made a gradual recovery with the normalization of the deep tendon reflexes and improvement in the eye droop. The histology report was suggestive of an AVM (Figure 3).

 

The patient has been on regular follow up in our clinic and has not developed any new symptoms.

## Discussion

The patient’s presentation as an extra-dural hematoma following rupture is an uncommon entity with a reported incidence of only 5 % among all spinal AVM's [1].In a recent study constituting more than 1000 cases of spinal epidural hematomas, the etiology behind the bleed was iatrogenic factors in 18% of the cases, non-iatrogenic factors in 29% of the cases, multifactorial in 11.1% of the cases whereas the cause was not reported in 42% of the cases [2].

We report the first case of an extradural spinal AVM in the cervico-thoracic junction presenting as Horner’s syndrome in a young male following its rupture.

Spinal AVM’s have been classified into various categories as per the distribution pattern of its feeding arteries and the draining veins [3].

The management of spinal vascular malformations depends on various factors such as their mode of presentation, the duration of clinical symptoms in patients, their radiological sub-types, and the availability of resources and skilled manpower [3].

Spinal AVM’s should always be kept as a differential diagnosis of a spinal hematoma in any cases presenting with myelopathy following a trivial injury and without any prior history of bleeding disorders. Early diagnosis and its emergent evacuation is the cornerstone in the management for a good outcome. It is always advisable to send the hematoma for further histopathologic study so as to not miss such entities behind the bleed.

Oldfield and Doppman had classified spinal vascular malformations into extradural, glomus, juvenile, and peri-medullary fistulas. Spetzler and colleagues also re-emphasized the fact to include the extra-dural variants into the classification of spinal vascular lesions as these lesions have the tendency to cause myelopathy owing to compression from venous channels, medullary venous congestion, vascular steal phenomenon, or from a resultant hemorrhage [3].

In such cases of extra-dural AVM’s, only surgical interruption of the arterio-venous shunt near the dural sleeves of the nerve is required for a good surgical outcome [4]. 

## Conclusions

A ruptured extradural AVM in the cervico-thoracic region can present initially as Horner's syndrome. Therefore, it is prudent to have early appropriate radio-imaging examinations followed by an emergent evacuation of the hematoma for a better functional outcome. It is also advisable to conduct a biopsy for the histological confirmation of occasional hidden entities such as AVM.
